# Multicenter study on the prevalence of colonization due to carbapenem-resistant *Enterobacterales* strains before and during the first year of COVID-19, Italy 2018–2020

**DOI:** 10.3389/fpubh.2023.1270924

**Published:** 2023-12-19

**Authors:** Teresa Fasciana, Alberto Antonelli, Gabriele Bianco, Donatella Lombardo, Giulia Codda, Emanuela Roscetto, Marianna Perez, Dario Lipari, Ignazio Arrigo, Elena Galia, Maria Rita Tricoli, Maddalena Calvo, Claudia Niccolai, Fabio Morecchiato, Giulia Errico, Stefania Stefani, Rossana Cavallo, Anna Marchese, Maria Rosaria Catania, Simone Ambretti, Gian Maria Rossolini, Annalisa Pantosti, Anna Teresa Palamara, Michela Sabbatucci, Nicola Serra, Anna Giammanco

**Affiliations:** ^1^Department of Health Promotion, Maternal and Child Health, Internal Medicine and Medical Specialies “G. D’Alessandro”, University of Palermo, Palermo, Italy; ^2^Department of Experimental and Clinical Medicine, University of Florence, Florence, Italy; ^3^Microbiology and Virology Unit, Careggi University Hospital, Florence, Italy; ^4^University Hospital Città della Salute e della Scienza di Torino, Turin, Italy; ^5^Unit of Microbiology, IRCCS Azienda Ospedaliero-Universitaria di Bologna, Bologna, Italy; ^6^Department of Surgical Sciences and Integrated Diagnostics (DISC), University of Genoa, Genova, Italy; ^7^Department of Molecular Medicine and Medical Biotechnology, University of Naples Federico II, Naples, Italy; ^8^Department of Biomedical and Biotechnological Sciences, University Hospital Policlinico Rodolico San Marco, Catania, Italy; ^9^Department of Infectious Diseases, Istituto Superiore di Sanità, Rome, Italy; ^10^Microbiology Unit, IRCCS Ospedale Policlinico San Martino, Genoa, Italy; ^11^Department of Public Health and Infectious Diseases, Sapienza University, Rome, Italy; ^12^Directorate General for Health Prevention, Ministry of Health, Rome, Italy; ^13^Department of Public Health, University Hospital Federico II, Naples, Italy

**Keywords:** *Enterobacterales*, Italy, antibiotic-resistance, carbapenem-resistant infections, prevalence, ICU, CRE, MDR

## Abstract

**Background:**

Among multidrug-resistant (MDR) bacteria able to threaten human health, carbapenem-resistant *Enterobacterales* (CRE) have become a major public health threat globally. National and international guidelines point out the importance of active routine surveillance policies to prevent CRE transmission. Therefore, defining lines of intervention and strategies capable of containing and controlling the spread of CRE is considered determinant. CRE screening is one of the main actions to curb transmission and control outbreaks, outlining the presence and also the prevalence and types of carbapenemase enzymes circulating locally.

**Objective:**

The purpose of this study was to outline the epidemiology of CRE colonization in Italy, detecting CRE-colonized patients at admission and during hospitalization, before and during the first year of COVID-19.

**Materials and methods:**

A total of 11,063 patients admitted to seven different hospitals (Bologna, Catania, Florence, Genoa, Naples, Palermo, and Turin) in Intensive Care Units (ICU) and other wards (non-ICU) located in the North, Center, and South of Italy were enrolled and screened for CRE carriage at admission (T0) and during the first 3 weeks of hospitalization (T1-T3). The study spanned two periods, before (September 2018-Septemeber 2019, I observational period) and during the COVID-19 pandemic (October 2019–September 2020, II observational period).

**Results:**

Overall, the prevalence of CRE-colonized patients at admission in ICU or in other ward, ranged from 3.9 to 11.5%, while a percentage from 5.1 to 15.5% of patients acquired CRE during hospital stay. There were large differences between the I and II period of study according to the different geographical areas and enrolling centers. Overall, comparison of prevalence of CRE-positive patients showed a significant increased trend between I and II observational periods both in ICU and non-ICU wards, mostly in the Southern participating centers. KPC-producing *Klebsiella pneumoniae* was the most frequent CRE species-carbapenemase combination reported in this study. In particular, the presence of KPC-producing *K. pneumoniae* was reported in period I during hospitalization in all the CRE-positive patients enrolled in ICU in Turin (North Italy), while in period II at admission in all the CRE-positive patients enrolled in ICU in Catania and in 58.3% of non-ICU CRE-positive patients in Naples (both centers in South Italy).

**Conclusion:**

The prevalence of CRE in Italy highly increased during the COVID-19 pandemic, mostly in the Southern hospital centers. KPC-producing *K. pneumoniae* was the most frequent colonizing CRE species reported. The results of our study confirmed the crucial value of active surveillance as well as the importance of multicenter studies representing diverse geographical areas even in endemic countries. Differences in CRE colonization prevalence among centers suggest the need for diversified and center-specific interventions as well as for strengthening efforts in infection prevention and control practices and policies.

## 1 Introduction

In the last decade, a dramatic and progressive increase in multidrug-resistant (MDR)-related infections occurred globally, mainly associated with carbapenem-resistant *Enterobacterales* (CRE), and in particular with *K. pneumoniae*, (carbapenem-resistant *K. pneumoniae*, CRKP). The frequency of *K. pneumoniae* human infections is determined by its ubiquitous presence in nature (1), and its better survival on surfaces than other *Enterobacterales.* CRKP has been associated with a higher number of hospital outbreaks than other *Enterobacterales*. Even if different mechanisms have been recognized as responsible for the carbapenem-resistant phenotype of CRE, other than carbapenemases production, the spread of CRE is mainly attributed to the high transmissibility of plasmid-mediated resistance determinants (2). Although *K. pneumoniae* is the most common CRE species recognized as a cause of serious infections, more frequently localized in the respiratory tract, urinary tract, and bloodstream, carbapenem resistance has also been increasingly reported in *Escherichia coli*, *Klebsiella oxytoca*, and *Enterobacter cloacae* (3, 4).

In endemic areas, the prevalence of CRE colonization in hospitalized patients ranges from 3 to 7% and it can be higher in patients admitted to intensive care units (ICU) (5).

*Enterobacterales*, including *K. pneumoniae*, can reside as colonizers in the human intestine, which is one of the potential endogenous reservoirs of CRE, followed by the oropharynx, skin, and urinary tract (6, 7). Because intestinal colonization by CRE tends to persist for long periods, this condition is considered one of the most determinant risk factors for infection (8, 9). To avoid these infections, associated with high morbidity and mortality (10, 11), the prevention of CRE colonization is considered crucial (9, 12). Recently, some clinical reports have presented local or national infection-control interventions (9) and strategies to decolonize CRE-positive subjects (13–15).

Even though the likelihood of infections among individuals colonized by CRE is variable, the early detection of the colonization status is considered relevant in preventing CRE spread (8, 9). Data on CRE colonization are considered necessary to guide decision-making regarding infection-control interventions such as the application of contact precautions and isolation of colonized patients (8).

Italy is considered an area of high endemicity for CRE, most of them represented by KPC-producing *K. pneumoniae.* The proportion of CRKP causing bloodstream infections raised dramatically in the last decade, from 1.3% in 2009 to 26.7% in 2011, peaking at 34.3% in 2013, then fluctuating between 26.8% in 2018, 29.5% in 2020 and 26.7% in 2021 (16, 17). Since 2011, the Ministry of Health recommended CRE screening the for carriage and provided indications for microbiological laboratories.

The aim of this study was to estimate the prevalence of CRE-colonized patients at the time of admission and the percentage of patients acquiring CRE during hospitalization in North, Central, and South Italy from September 2018 to September 2020, highlighting geographic and local diversity. This is the largest Italian multicenter study aiming to identify CRE carriers and to describe the epidemiology of CRE colonization in Italy.

## 2 Materials and methods

### 2.1 Study design

The project was approved by the National Center for Disease Prevention and Control (CCM) of the Italian Ministry of Health in 2017, to run from February 2018 to February 2019. Following the SARS-CoV-2 emergency, it was extended until January 2021 and consequently, the enrolment of patients was carried out from September 2018 to September 2019 (I observational period) and from October 2019 to September 2020 (II observational period).

The objectives of the project were to provide an overview of the microbiological methods in use at 7 bacteriological centers (Bologna, Catania, Florence, Genoa, Naples, Palermo, and Turin) distributed throughout the country, and to provide an estimate of the prevalence of CRE colonization in the hospital setting, in order to draft national recommendations.

For data collection, a dedicated IT platform was supplied to upload center information, patients’ demographic and clinical characteristics, including microbiological data, and the colonization status. In each center, the project included all patients admitted to ICU and whenever possible, patients admitted to Units of Surgery (US), Hematology (UH), and Medicine (UM).

### 2.2 Participating centers

Seven hospital centers that geographically represented North, Center, and South Italy were included in the study (North: University Hospital Città della Salute e della Scienza di Torino, Turin; University Hospital San Martino, Genoa;University Hospital S. Orsola-Malpighi, Bologna; Center: Careggi University Hospital, Florence. South: University Hospital Federico II, Naples; University Hospital Policlinico Rodolico-San Marco, Catania; University Hospital P. Giaccone, Palermo). It also involved the Department of Infectious Diseases, Istituto Superiore di Sanità (ISS) Roma for scientific support, and the Department ASOE of the Sicilian Region, responsible for administration procedures.

### 2.3 Sample collection

Samples (rectal swabs) for CRE screening were obtained from all patients (including those transferred from other hospitals or re-admitted to the same hospital) on admission (T0). In addition, CRE screening was repeated from patients negative on admission weekly for a maximum of 3 weeks (time points T1, T2, and T3). If CRE-positive, patients were excluded from the subsequent screening.

### 2.4 Microbiological procedures for CRE screening

Specimens were processed by direct plating into selective chromogenic agar plates (culture-based method). After 24 h, the colonies growing on agar plates (BBL CHROMagar CPE,Becton Dickson, or ChromID Carba Smart, bioMérieux, France), were identified by automatic systems: Phoenix (BD, New Jersey, United States) and/or Vitek2 (bioMérieux) and/or MALDI –TOF MS (Bruker, Massachusetts, United States). Confirmed CRE were further screened by molecular methods (GeneXpert – Cepheid System Software or Allplex EnteroDR assay, Seegene, Republic of Korea) to identify the most common carbapemenase genes: *bla*_KPC_, *bla*_IMP_, *bla*_VIM_, *bla*_NDM_, and *bla*_OXA-48_-like. The presence of other less representative carbapenemases was not investigated.

### 2.5 Statistical analysis

Data are presented as numbers and percentages for categorical variables and continuous data are expressed as mean ± standard deviation (SD) unless otherwise specified.

The multi-comparison chi-square test was used to define significant differences among groups, if the chi-square test was positive (value of *p* <0.05) then residual analysis with *Z*-test was performed to localize the highest or lowest significant presence.

All tests with *p* < 0.05 were considered significant. All data were analyzed with Matlab statistical toolbox version 2008 (Math Works, Natick, MA, United States) for 32-bit Windows.

## 3 Results

### 3.1 Samples collection

A total of 15,630 rectal swabs were collected from 11,063 patients enrolled from 1 September 2018 to 1 September 2020; particularly, 2,958 patients from Florence, 1,902 from Palermo, 1,816 from Bologna, 1,416 patients from Turin, 1,247 from Naples, 1,027 from Genoa, and 697 from Catania. In particular, 7,268 patients (with 10,101 swabs) were enrolled during the first study period (period I: September 2018–September 2019) and 3,795 patients (with 5,529 swabs) during the second period (period II: October 2019–September 2020).

The numbers of colonized patients during the two observational periods are detailed in Table 1 distributed by hospitalization center, ward (ICU and non-ICU), observation period (I and II), and screening time (T0 and T1-T3). Overall, the prevalence of colonized patients was higher in period II than in period I, both for ICU than for non-ICU wards. Similarly, the proportion of patients that acquired CRE during hospitalization was higher in period II. However, the situation in the seven centers differed greatly and in some centers of North Italy an opposite trend was apparent.

**Table 1:** Patients screened at admission (T0) and during hospitalization (T1-T3), distributed in the various geographical centres, in intensive care unit (ICU) or other wards (non-ICU), over the two observational periods, assessing the prevalence of colonization due to carbapenem-resistant enterobacterales (CRE) strains.

**Centres**	**ICU**				**Non-ICU**			
	Period I		Period II		Period I		Period II	
	T0	T1-T3	T0	T1-T3	T0	T1-T3	T0	T1-T3
Genoa Patients Positive patients	370 16 (4.3%)	325 21 (6.5%)	211 7 (3.3%)	180 3 (1.7%)	251 19 (7.6%)	196 12 (6.1%)	195 10 (5.1%)	173 3 (1.7%)
Turin Patients Positive patients	1024 31 (3.0%)	170 59 (34.7%)	392 13 (3.3%)	84 17 (20.2%)	—	—	—	—
Bologna Patients Positive patients	515 41 (8.0%)	427 42 (9.8%)	244 14 (5.7%)	230 15 (6.5%)	834 31 (3.7%)	420 21 (5.0%)	223 11 (4.9%)	210 15 (7.1%)
Florence Patients Positive patients	2157 69 (3.2%)	438 33 (7.5%)	402 17 (4.2%)	90 5 (5.6%)	325 18 (5.5%)	165 2 (1.2%)	74 7 (9.5%)	47 6 (12.8%)
Catania Patients Positive patients	200 5 (2.5%)	115 12 (10.4%)	122 54 (44.3%)	65 2 (3.1%)	213 3 (1.4%)	89 3 (3.4%)	162 70 (43.2%)	86 8 (9.3%)
Naples Patients Positive patients	168 6 (3.6%)	78 5 (6.4%)	164 14 (8.5%)	62 12 (19.4%)	494 10 (2.0%)	171 3 (1.8%)	421 12 (2.9%)	125 6 (4.8%)
Palermo Patients Positive patients	465 49 (10.5%)	165 45 (27.3%)	489 113 (23.1%)	142 78 (54.9%)	252 12 (4.8%)	74 16 (21.6%)	696 85 (12.2%)	240 48 (20.0%)
Total Patients Positive patients	4899 217 (4.4%)	1718 217 (12.6%)	2024 232 (11.5%)	853 132 (15.5%)	2369 93 (3.9%)	1115 57 (5.1%)	1771 195 (11.0%)	881 86 (9.8%)

Italy 2018–2020.

### 3.2 CRE-colonized patients in ICU

In ICU the highest percentages of colonized patients at admission were found in the southern centers, Palermo and Catania in both periods but especially in the second period with 23.1 and 44.3% of colonized patients, respectively, (Table 1). During hospitalization, the percentage of patients who became colonized with CRE was high in all centers in both periods (>X%): in the first period the highest percentage of patients who acquired CRE during hospitalization was observed in Turin (34.7%), in the second period in Palermo (54.9%).

### 3.3 CRE-colonized patients in non-ICU

In the first period in non-ICU wards, the prevalence of colonized patients at admission was low in all centers with the highest percentage in Genoa (7.6%). During hospitalization, a further percentage of patients who were negative at admission, acquired CRE. The percentage was the highest in Palermo (21.6%). In the second period, the highest prevalence of colonized patients on admission was found in Catania (43.2%), and the highest percentage of patients colonized during hospitalization in Palermo (20.0%).

### 3.4 Comparison of colonized patients between ICU and non-ICU

**Figure 1:**
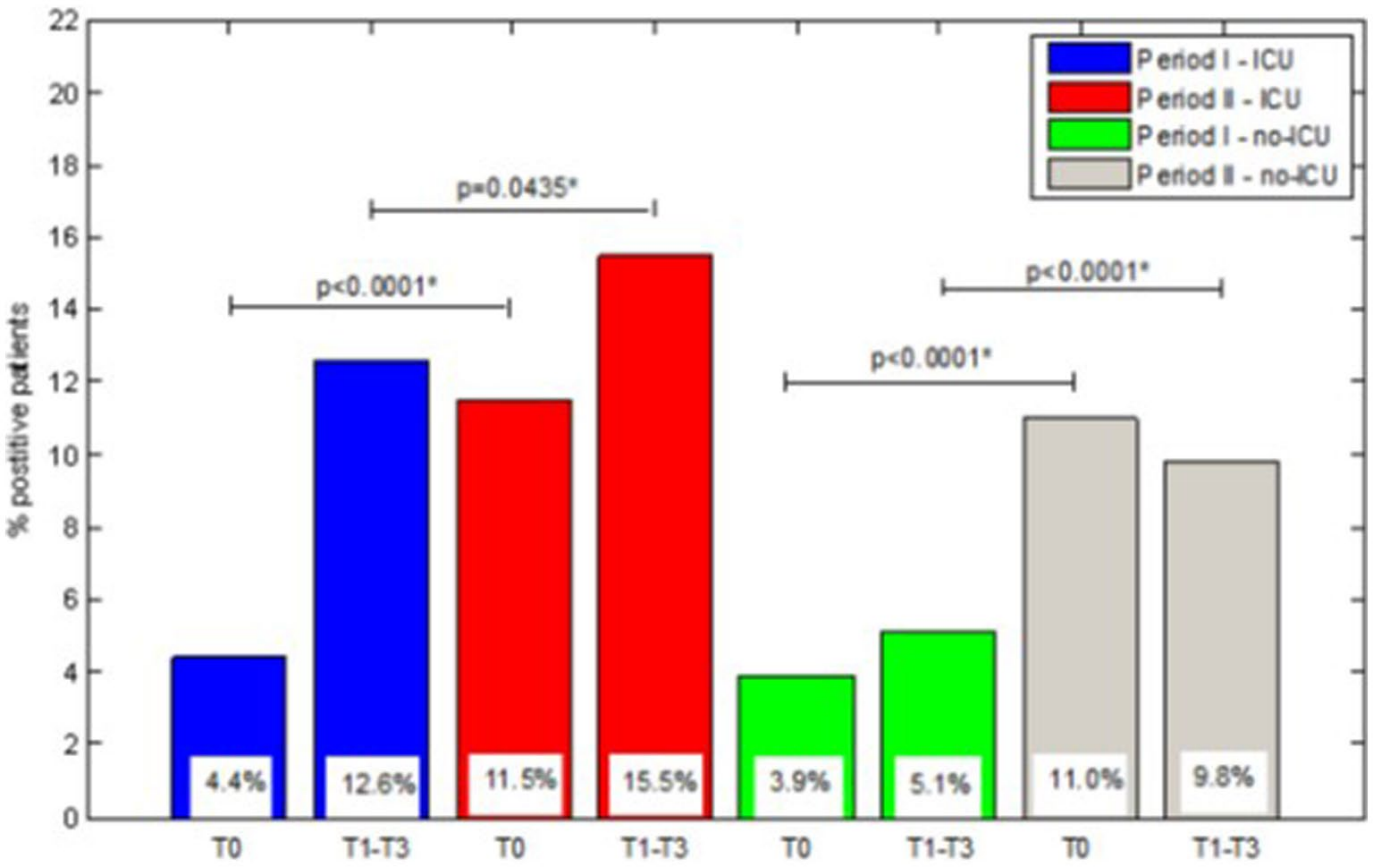
Comparision of percentage of CRE-positive patients between ICU and no-ICU wards over I and II starvation periods.

In Figure 1, the percentages of colonized patients on admission and the percentages of patients who acquired colonization during hospital stay in ICU and non-ICU wards were compared between the two-observation period. The difference between periods I and II was significant for both colonization at admission and acquired colonization in ICU and also in non-ICU wards.

### 3.5 Microbiological characterizations of CRE isolates

Among CRE isolates from the colonized patients, KPC-producing *K. pneumoniae* was the most frequent species/carbapenemase combination identified throughout the study (Supplementary Tables S1–S4). *E. coli* and *Enterobacter* spp. were occasionally recovered in several centers from patients at admission or during hospitalization. Occurrence of isolates carrying multiple carbapenemase genes was observed among CRE isolates from ICU and non-ICU patients admitted at Bologna, Florence and Palermo centers. These isolates harbored *bla*_KPC_ in association with another carbapenemase gene (*bla*_VIM_, *bla*_NDM_, or *bla*_OXA-48-like_). In a few CRE isolates no carbapenemase gene was detected. Resistance mechanisms different from carbapenemase can be suggested for a few *Enterobacter* spp. isolates from Genoa and Naples and for *E. coli*, *Citrobacter* spp. and *K. pneumoniae* from Naples and Palermo (Supplementary Tables S1–S4). The analytical description of the prevalence of carbapenemase negative strains is shown in Supplementary Figure S1.

### 3.6 Statistical analysis of data related to colonization due to CRE

**Figure 2:**
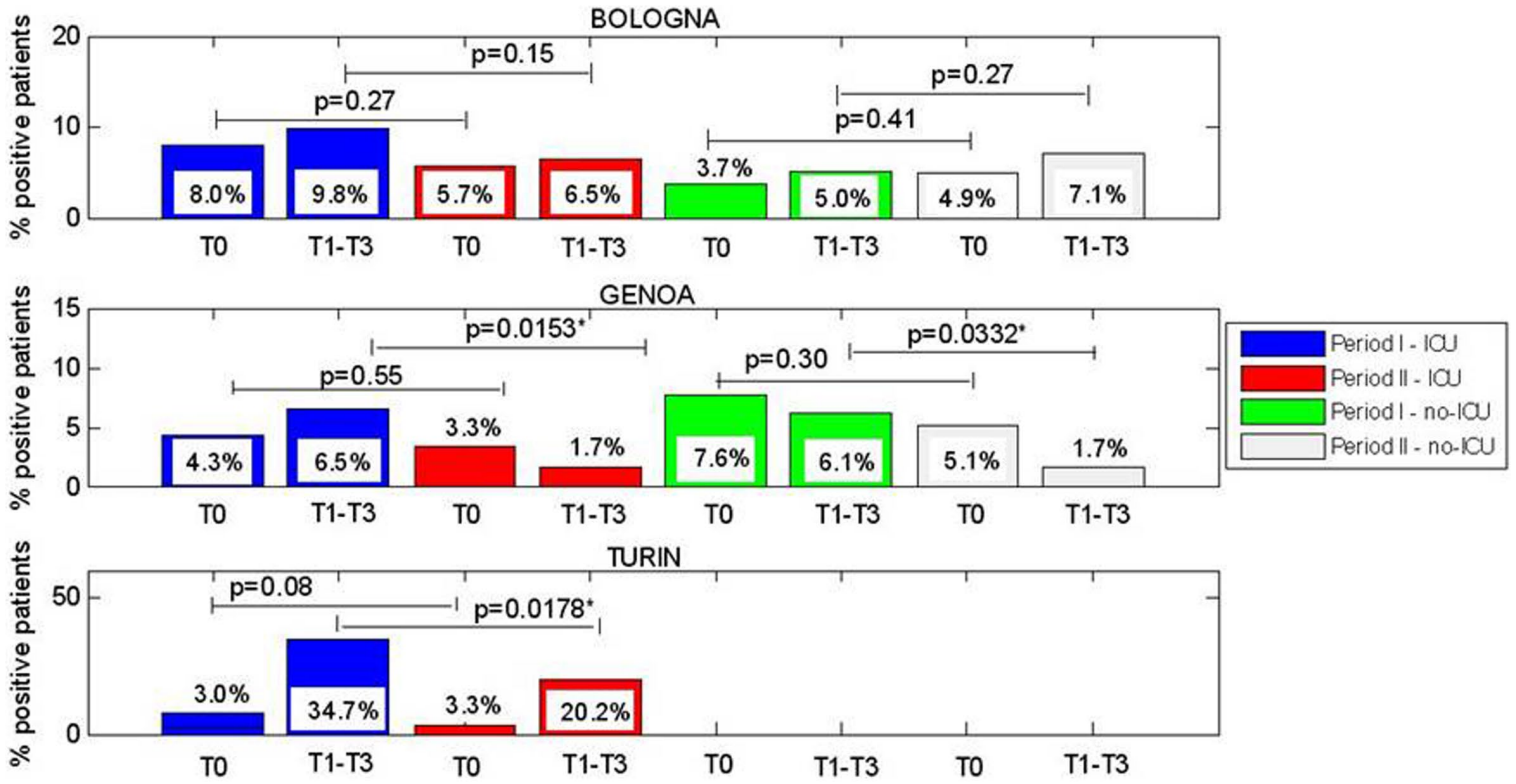
**(A–C)** Comparision of CRE positive patients between Period I and Period II at admission (T0), and between week1 and week 3 of hospitalization (T1-T3), for ICU wards and considering centres located in north Italy.

**Figure 3:**
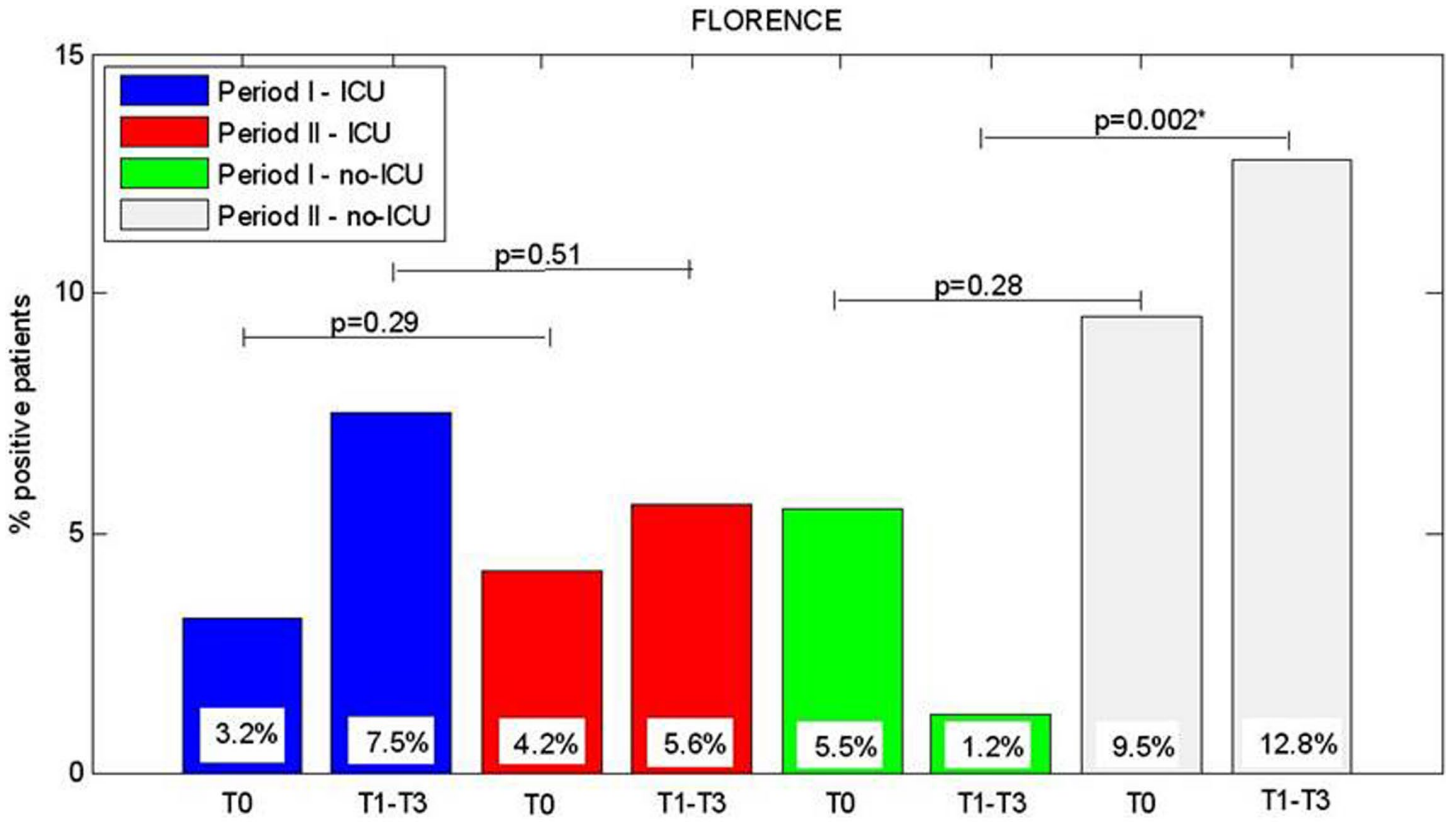
Comparision of CRE positive patients between Period I and Period II and T0, admission, T1-T3 hospitalization period, for ICU Opertive Units and considering the Hospital centre allocated in Central Italy.

**Figure 4:**
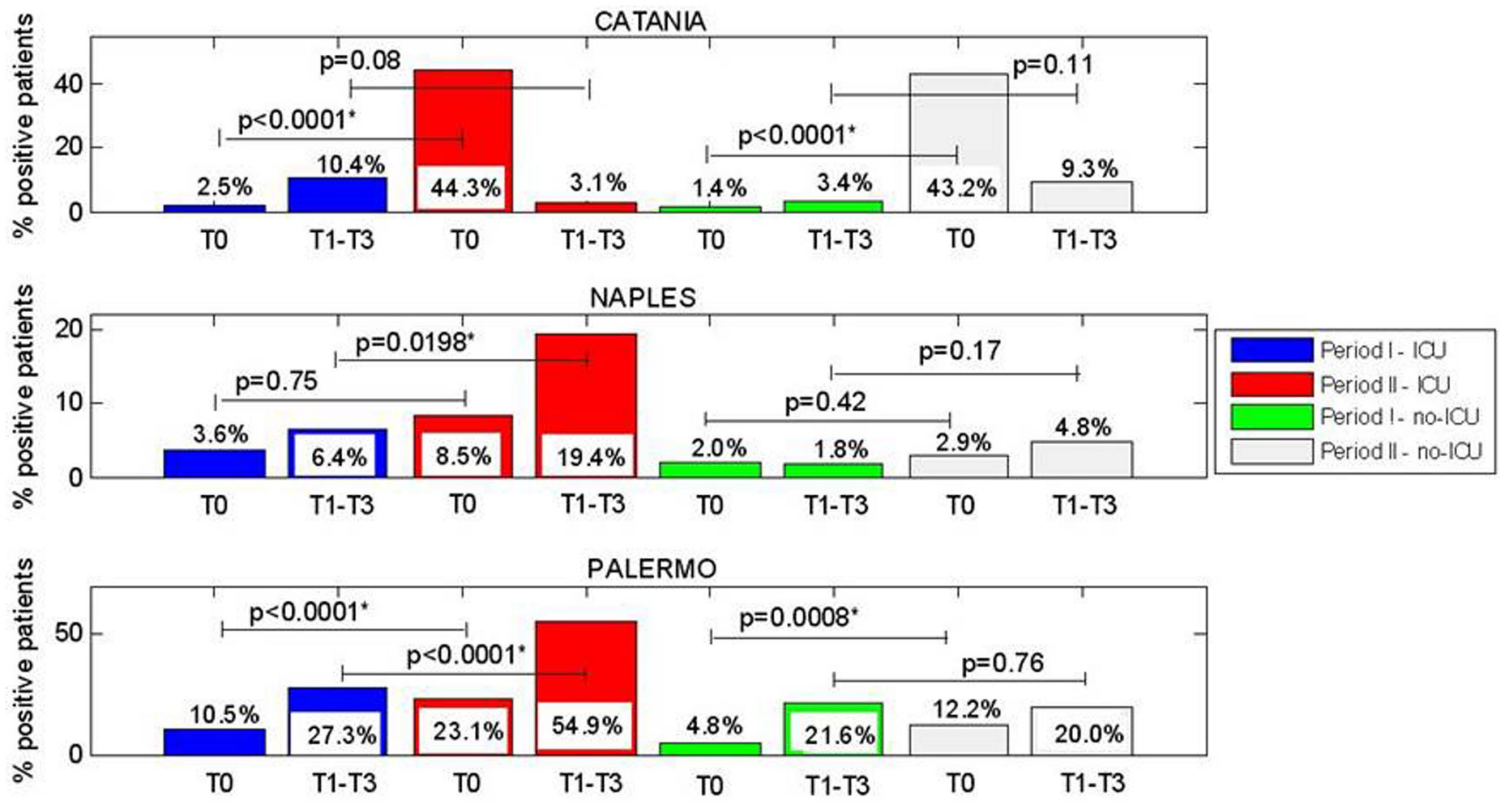
**(A–C)** Comparision of CRE positive patients between Period I and Period II at admission (T0), and between week1 and week 3 of hospitalization (T1-T3), for ICU and no-ICU wards and considering centres located in South Italy.

We analyzed the significant difference between rates of CRE-positive patients at each control points in a single period or considering the two periods in ICU and non-ICU units. Briefly, we stratified the patients who resulted colonized by Hospital Center and geographic area (North, Center, and South of Italy). The results are reported in Figures 2–4.

About Northern Italy, for Bologna, no significant differences were observed. For Genoa, significant differences were observed between periods I and II for patients who acquired CRE during hospitalization (T1-T3) with lower rates in the period II in both ICU (6.5% vs. 1.7%, *p* = 0.0153) and non-ICU units (6.1% vs. 1.7%, *p* = 0.0332); this was also observed for Turin in ICU (34.7% vs. 20.2%, *p* = 0.0178).

Regarding Central Italy (Florence center), for non-ICU wards at T1-T3 a difference between periods I and II were observed (1.2% vs. 12.8%, *p* = 0.002) with a significant increase of patients who became colonized during hospitalization.

Regarding Southern Italy, for Catania, a very high increase in the percentage of patients who were colonized at admission (T0), between period I and II for ICU (2.5 vs. 44.3%, *p* < 0.0001) and non-ICU wards (1.4% vs. 43.2%, *p* < 0.0001) was observed.

For Naples, we found in ICU a significant increase between the percentage of patients who acquired CRE in T1-T3 between period I and II and (6.4% vs. 19.4%, *p* = 0.0198). For non-ICU, no significant differences were observed.

For Palermo, a significant increase between period I and II in the percentage of patients who were colonized at admission at T0 (10.5% vs. 23.1%, *p* < 0.0001) and also in the percentage of patients who acquired CRE during hospital stay at T1-T3 (27.3% vs. 54.9%, *p* < 0.0001) was observed in ICU. In non-ICU we found a significant difference, between periods I and II, only for the prevalence of patients colonized at admission (4.8% vs. 12.2%, *p* = 0.0008).

Additional analysis were reported in Supplementary Table S5, where statistical analysis of the proportion of CRE strains isolated at the various centres in Intensive Care Unit (ICU) and other wards was reported.

## 4 Discussion

Since 2010, in Italy, the spread of CRE increased rapidly with numerous outbreaks in acute care hospitals (18–20) and in rehabilitation facilities (21, 22). Currently, CRE, especially KPC-producing *K. pneumoniae* strains (2), are considered endemic in the country (19) and the phenomenon heavily affects social and economic costs. It is, therefore, a priority to identify, evaluate, and define lines of intervention and strategies capable of containing and controlling the spread of CRE. The implementation of a surveillance system that quantifies the problem and makes microbiological data available, for epidemiological purposes, is considered relevant (2, 19). Also, it is important to relate the CRE spread to the quality of the microbiological diagnosis, to the indications for treatment of infections and to the type of empirical and/or targeted antibiotic chosen.

In Italy, colonization with CRE ranges from 0.2 to 3.9% in different settings (23, 24) and is associated with a 16.5% overall risk of infection (8). In addition, intestinal colonization by CRE once established tends to persist for long periods of time (14), and colonized subjects represent the main source of contamination for the intra-hospital diffusion of CRE, also favoring the interspecies transmission of resistance genes (15). In 2016, the ECDC reported actions to reduce the risk of CRE infections emphasizing the activities of screening/surveillance to prevent transmission of carbapenemase-production bacteria (18). On the bases of these considerations, knowledge of CRE colonization is considered relevant not only to limit transmission but also for controlling infection, especially in high-risk patients.

This study aimed to provide data on the prevalence of patients colonized by CRE in seven Italian centers, and on the role of hospitalization in the acquisition of CRE colonization. For this purpose, patients were screened at admission and during hospitalization for the presence of CRE and other carbapenemases besides *bla*_KPC_, such as *bla*_VIM_, *bla*_NDM_, and *bla*_OXA-48-_like, which although less common, can contribute to the spread of CRE (9).

For the screening of CRE rectal carriage over the study period, the relevance of the protocol based on the use of selective chromogenic media followed by carbapenemase genes investigation by molecular testing was confirmed. The advantage of using molecular assays to screen carbapenemase genes is obviously related to faster turnaround time and higher sensitivity including the ability to detect carbapenemases with weak or impaired activity such as KPC variants associated with ceftazidime/avibactam resistance (25). However, because molecular testing has higher costs than phenotypic detection methods, for many laboratories it is considered a second-level test that cannot be used in large numbers. Of note, the rapid detection of CRE-carriers by molecular tests would allow advance in CRE surveillance and infection control with several improvements on prevalence rates, length of hospital stay, workload for the healthcare staff, and overall hospital costs indirectly.

On the bases of this experience, although the proposal of a national guideline for the screening of CRE-positive patients at hospital admission could be useful, its wide application might require considerable efforts. However, the use of molecular tests is recommended for a quick identification of CRE carriers which would allow an improvement in infection control practices with consequences on prevalence rates and indirectly on hospital costs.

The results obtained from the evaluations performed demonstrated the greater presence of CRE stains in South Italy, that are known to be associated with new emerging *K. pneumoniae* clones (26–28). As already observed, the presence of a higher percentage of CRE-positive patients is related to hospitalization in ICU, where at-risk patients are prone to acquire invasive CRE infections (19).

Concerning the epidemiology of CRE, the data confirmed KPC-producing *K. pneumoniae* to be the most common CRE circulating in Italy and this characteristic was not influenced by the COVID-19 pandemic (Supplementary Tables S1–S4). The rate of KPC-producing *K. pneumoniae* did not increase significantly during hospitalization and strains characterized by more than one carbapenemase were observed mainly in Florence and Palermo. Reports regarding occurrence of multiple carbapenemases producers among *Enterobacterales* is increasing in Italy and worldwide, with dual carbapenemase-producing strains such as *bla*_VIM_, *bla*_NDM_, and *bla*_OXA-48-_like, *bla*_KPC_ + *bla*_VIM_, *bla*_KPC_ + *bla*_NDM_, *bla*_OXA-48-_like+*bla*_NDM_, *bla*_OXA-48-_likes+*bla*_VIM_ (29–37). Carbapenemases of *bla*_VIM_ and *bla*_NDM_ types in *Enterobacterales*, isolated for the first time in 2004 (29) and 2012 (30) respectively, were still present, according to the results obtained. Although MBL producers have been constantly detected for years, they are still sporadically isolated in Italy with the exception of a prolonged outbreak by NDM-producing *K. pneumoniae* ongoing in Tuscany since 2018, which has now become endemic (31).

The results of this study underline the importance of active surveillance and the need to strengthen infection control measures. While in the period II of the study (COVID-19 period) in the centers of South Italy there was an increase in the prevalence of CRE carriers, both at admission and during hospitalization, in North Italy there were no relevant changes. The lower prevalence of CRE carriers in North Italy is in line with the lower percentage of carbapenem-resistant *K. pneumoniae* among isolates causing bloodstream infections in regions of North Italy compared to regions of Center and South Italy, as reported by the National Surveillance of antibiotic-resistance AR-ISS (32). The lower prevalence of CRE-positive patients in Northern Italy compared to other areas may be due to a higher attention to control measures at the hospital level. In fact, a multi-modal approach including active screening for CRE, antimicrobial stewardship and infection control actions (19, 22–24) was adopted in several hospitals of North Italy and likely contributed to limit the diffusion of CRE [Especially the rapid and accurate identification of colonized patients is essential to decrease the diffusion of CRE, since carriage is predictive for CRE bloodstream infections (24)].

Overall, our national data showed that during the COVID-19 pandemic (data registered in period II) the prevalence of CRE increased significantly both at admission and during hospitalization, in ICU as well as in non-ICU (38, 39). This was not observed in some centers separately, i.e., Genoa and Turin in the North, where a decreasing trend was registered. Besides differences in the adoption of infection control measures, the different trends between North and South Italy can be due also to the different characteristics of patients hospitalized during the COVID-19 pandemic (mainly community patients) reflecting the different impact of the pandemic through the geographical areas, different management of patients related to high intensity of care, and different organization of the health care structures (31, 38).

Our study has some limitations. One important limitation is that some participating center had already in place an active surveillance of CRE, while others started the screening of patients when this project was initiated. Four centers out of seven applied direct molecular methods to detect CRE carriage. In addition, it was not possible to distinguish a new patient admission from a transfer from a different ward or a different hospital or another setting such as a long-term care facility. We have no information on the follow up of CRE-positive patients, including information on deaths, or on the COVID-19 status.

In conclusion, we stress the need for continuous active surveillance of CRE in Italy including screening for CRE colonization.

## Data availability statement

The raw data supporting the conclusions of this article will be made available by the authors, without undue reservation.

## Ethics statement

The studies involving humans were approved by the Ethics Committee was obtained by the Azienda Ospedaliera Universitaria Policlinico “P. Giaccone” of Palermo (protocols n°07/2019). The studies were conducted in accordance with the local legislation and institutional requirements. Written informed consent for participation was not required from the participants or the participants’ legal guardians/next of kin because all data used in the study were anonymized, according to the requirements set by the Italian Data Protection Code (leg. Decree 196/2003) and by the general authorizations issued by the Data Protection Authority. Written informed consent was not obtained from the individual(s) for the publication of any potentially identifiable images or data included in this article because samples are routinely evaluated for CRE and data collected are anonymous.

## Author contributions

TF: Data curation, Writing – original draft. AA: Data curation, Writing – original draft. GB: Data curation, Writing – original draft. DoL: Data curation, Writing – original draft. GC: Data curation, Writing – original draft. ER: Data curation, Writing – original draft. MP: Data curation, Writing – original draft. DaL: Data curation, Writing – original draft. IA: Data curation, Writing – original draft. EG: Data curation, Writing – original draft. MRT: Data curation, Writing – original draft. MadC: Data curation, Writing – original draft. CN: Data curation, Writing – original draft. FM: Data curation, Writing – original draft. GE: Writing – original draft, Investigation. SS: Conceptualization, Investigation, Methodology, Supervision, Writing – original draft. RC: Investigation, Methodology, Writing – review & editing. AM: Investigation, Methodology, Writing-original draft. MarC: Investigation, Methodology, Writing – original draft. SA: Data curation, Investigation, Writing – original draft. GR: Conceptualization, Investigation, Methodology, Supervision, Writing original draft. AP: Investigation, Methodology, Supervision, Writing review & editing. ATP: Conceptualization, Supervision, Funding acquisition, Writing review & editing. MS: Investigation, Supervision, Writing – review & editing, Project administration. NS: Investigation, Writing – review& editing, Formal analysis. AG: Writing – review & editing, Conceptualization, Funding acquisition, Methodology, Supervision & editing, Formal analysis.

## Collaborative working group

Scientific consultant: Tommaso Giani (Department of Experimental and Clinical Medicine, University of Florence, Florence, Italy. Microbiology and Virology Unit, Careggi University Hospital, Florence, Italy); contributors to data collection: Lara Mosconia (Department of Experimental and Clinical Medicine, University of Florence, Florence, Italy; Microbiology and Virology Unit, Careggi University Hospital, Florence, Italy.), Eleonora Riccobono (Department of Experimental and Clinical Medicine, University of Florence, Florence, Italy. Department of Medical Biotechnologies, University of Siena), Alessandro Bondi and Marco Peradotto (University Hospital Città della Salute e della Scienza di Torino, Turin, Italy).
