# Supplementary material for: Multicenter study on the prevalence of colonization due to carbapenem-resistant Enterobacterales strains before and during the first year of COVID-19, Italy 2018–2020
Source: Front Public Health. 2023 Dec 19;11:1270924. doi: 10.3389/fpubh.2023.1270924 (PMC10771343; doi:10.3389/fpubh.2023.1270924)
Supplement: Supplementary file 1 [file Table_1.DOC]

# Supplementary material

# Supplementary Table 1-5

**Table 1.** Prevalence of carbapenem-resistant *Enterobacterales* (CRE) strains isolated in the various centres in Intensive Care Unit (ICU) in the observation period I at hospital admission (T0) and between week 1 and week 3 (T1-T3), Italy 2018-2019

| **Period I** | **T0** | | **T1 – T3** | |
| --- | --- | --- | --- | --- |
| **ICU** | **Bacteria** | **Gene** | **Bacteria** | **Gene** |
| GENOA  (16 positives at T0)  (21 positives at T1-T3) | 75.0%, *K. pneumoniae*  12.5%, *Enterobacter* spp.  6.3%, *Enterobacter* spp.  6.3%, *Enterobacter* spp. | *bla*_KPC_  *bla*_VIM_  *bla*_KPC_  *none* | 81.0%, *K. pneumoniae*  14.3%, *Enterobacter* spp.  4.8%, *Enterobacter* spp. | *bla*_KPC_  *none*  *bla*_KPC_ |
| TOURIN  (31 positives at T0)  (59 positives at T1-T3) | 93.5%, *K. pneumoniae*  3.2%, *E. coli*  3.2%, *Enterobacter* spp. | *bla*_KPC_  *bla*_KPC_  *bla*_VIM_ | 100%, *K. pneumoniae* | *bla*_KPC_ |
| BOLOGNA  (41 positives at T0)  (42 positives at T1-T3) | 87.8%, *K. pneumoniae*  4.9%, *E. coli*  2.4%, *K. pneumoniae*  2.4%, *K. pneumoniae*  2.4%, *K. pneumonia* | *bla*_KPC_  *bla*_NDM_  *bla*_NDM_  *bla*_VIM_  *bla*_OXA-48_-like | 71.4%, *K. pneumoniae*  23.8%, *K. pneumoniae*  2.4%, *K. pneumoniae*  2.4%, *K. pneumoniae* | *bla*_KPC_  *bla*_NDM_  *bla*_KPC_; *bla*_NDM_  *bla*_KPC_; *bla*_VIM_ |
| FLORENCE  (69 positives at T0)  (33 positives at T1-T3) | *79.7%, K. pneumoniae*  *10.1%, K. pneumoniae*  *2.9%, K. pneumoniae*  *1.5%, K. pneumoniae*  *1.5%, E. coli*  *1.5%, E. coli*  *1.5%, E. coli*  *1.5%, Enterobacter* spp. | *bla*_KPC_  *bla*_NDM_  *bla*_KPC_  *bla*_VIM_  *bla*_VIM_  *bla*_KPC_  *bla*_NDM_  *bla*_VIM_  *bla*_KPC_ | 72.7%, *K. pneumoniae*  9.1%, *K. pneumoniae*  6.1%, *K. pneumonite*  6.1%, *K. pneumoniae*  3.0%, *E. coli*  3.0%, *Enterobacter* spp. | *bla*_KPC_  *bla*_NDM_  *bla*_VIM_  *bla*_KPC_*; bla*_VIM_  *bla*_NDM_  *bla*_VIM_ |
| CATANIA  (5 positives at T0)  (12 positives at T1-T3) | 100%, *K.pneumoniae* | *bla*_KPC_ | 100%, *K. pneumoniae* | *bla*_KPC_ |
| NAPLES  (6 positives at T0)  (5 positives at T1-T3) | 66.7%, *K. pneumoniae*  16.7%*,* *K. pneumoniae*  16.7%*,**Enterobacter* spp. | *bla*_KPC_  *bla*_OXA-48_-like  *none* | 60.0%, *K. pneumoniae*  20.0%, *K. pneumoniae*  20.0%, *Enterobacter* spp. | *bla*_KPC_  *bla*_OXA-48_-like *bla*_KPC_ |
| PALERMO  (49 positives at T0)  (45 positives at T1-T3) | 75.5%, *K. pneumoniae*  8.2%, *E. coli*  6.1%**,** *K. pneumoniae*  4.1%, *Enterobacter* spp.  4.1%, *K.pneumoniae*  2.0%, *K. pneumoniae* | *bla*_KPC_  *bla*_KPC_  *bla*_KPC_*; bla*_OXA-48_-like  *bla*_VIM_  *bla*_OXA-48_-like  *bla*_NDM_ | 86.7%, *K. pneumoniae*  4.4%, *Enterobacter* spp.  4.4%, *K. pneumoniae*  2.2%, *K. pneumoniae*  2.2%, *E. coli* | *bla*_KPC_  *bla*_VIM_  *bla*_KPC_*; bla*_VIM_  *bla*_OXA-48_-like *bla*_KPC_ |

**Table 2.** Prevalence of carbapenem-resistant *Enterobacterales* (CRE) strains isolated in the various centres in Intensive Care Unit (ICU) in the observation period II at hospital admission (T0) and between week 1 and week 3 (T1-T3), Italy 2019-2020

| **Period II** | **T0** | | **T1 – T3** | |
| --- | --- | --- | --- | --- |
| **ICU** | **Bacteria** | **Gene** | **Bacteria** | **Gene** |
| GENOA  (7 positives at T0)  (3 positives at T1-T3) | 100%, *K. pneumoniae* | *bla*_KPC_ | 66.7%, *K. pneumoniae*  33.3% *Enterobacter* spp. | *bla*_KPC_  *bla*_KPC_ |
| TURIN  (13 positives at T0)  (17 positives at T1-T3) | 92.3%, *K. pneumoniae*  7.7%, *Enterobacter* spp. | *bla*_KPC_  *bla*_VIM_ | 88.2%, *K. pneumoniae*  5.9%, *E.coli*  5.9%, *Enterobacter* spp. | *bla*_KPC_  *bla*_KPC_  *bla*_VIM_ |
| BOLOGNA  (14 positives at T0)  (15 positives at T1-T3) | 71.4%, *K. pneumoniae*  14.3%, *K. pneumoniae*  7.1%, *K. pneumoniae*  7.1%, *Enterobacter* spp. | *bla*_KPC_  *bla*_NDM_  *bla*_VIM_  *bla*_KPC_ | 73.3%, *K. pneumoniae*  13.3%, *Enterobacter* spp.  5.7%, *K. pneumoniae*  6.7%, *K. pneumoniae* | *bla*_KPC_  *bla*_OXA-48_-like  *bla*_OXA-48_-like *bla*_VIM_ |
| FLORENCE  (17 positives at T0)  ( 5 positives at T1-T3) | 70.6%, *K. pneumoniae*  5.9%, *K. pneumoniae*  5.9%, *K. pneumoniae*  5.9%, *E. coli*  5.9%, *E. coli*  5.9%, *E. coli* | *bla*_KPC_  *bla*_NDM_  *bla*_KPC_*;bla*_VIM_  *bla*_KPC_  *bla*_OXA-48_-like  *bla*_KPC_*; bla*_VIM_ | 80.0%, *K. pneumoniae*  20.0%, *K. pneumoniae* | *bla*_KPC_  *bla*_KPC_ |
| CATANIA  (54 positives at T0)  ( 2 positives at T1-T3) | 100%(54), *K. pneumoniae* | *bla*_KPC_ | 100%, *K. pneumoniae* | *bla*_KPC_ |
| NAPLES  (14 positives at T0)  (12 positives at T1-T3) | 64.3%(9), *K. pneumoniae*  14.3%(2)*,* *K. pneumoniae*  14.3%(2), *Enterobacter* spp.  7.1%(1), *Enterobacter* spp. | *bla*_KPC_  *bla*_OXA-48_-like  *none*  *bla*_NDM_ | 66.7%, *K. pneumoniae*  8.3%, *K. pneumoniae*  8.3%, *K. pneumoniae*  8.3%, *E. coli*  8.3%, *Enterobacter* spp. | *bla*_KPC_  *bla*_OXA-48_-like  *bla*_VIM_  *bla*_KPC_  *bla*_KPC_ |
| PALERMO  (113 positive at T0)  ( 78 positives at T1-T3) | 86.7%(98), *K. pneumoniae*  2.7%(3), *Enterobacter* spp.  2.7%(3**),** *K. pneumoniae*  1.8%(2), *Enterobacter* spp.  1.8%(2), *K. pneumoniae*  1.8%(2), *K. pneumoniae*  0.9%(1), *K. pneumoniae*  0.9%(1), *E. coli*  0.9%(1), *E. coli* | *bla*_KPC_  *bla* _VIM_  *bla*_OXA-48_-like  *bla* _NDM_  *bla*_KPC_*; bla*_OXA-48_-like  *bla*_KPC_  *bla* _VIM_  *bla*_KPC_*; bla* _NDM_  *bla*_KPC_  *bla*_OXA-48_-like | 80.8%, *K. pneumoniae*  16.7%, *K. pneumoniae*  1.3%, *K. pneumoniae*  1.3%, *E. coli* | *bla*_KPC_  *bla*_KPC_*; bla* _NDM_ *bla*_OXA-48_-like  *bla*_KPC_ |

**Table 3.** Prevalence of carbapenem-resistant *Enterobacterales* (CRE) strains isolated in the various centres in wards different from Intensive Care Unit (non-ICU) in the observation period I at hospital admission (T0) and between week 1 and week 3 (T1-T3), Italy 2018-2019.

| **Period I** | **T0** | | **T1 – T3** | |
| --- | --- | --- | --- | --- |
| **no-ICU** | **Bacteria** | **Gene** | **Bacteria** | **Gene** |
| GENOA  (19 positives at T0)  (12 positives at T1-T3) | 78.9%, *K. pneumoniae*  5.3%, *E. coli*  5.3%, *Enterobacter* spp.  5.3%, *Enterobacter* spp.  5.3%, *Enterobacter* spp. | *bla*_KPC_  *bla*_NDM_  *bla*_KPC_  *bla*_VIM_  *none* | 75.0%, *K. pneumoniae*  16.7%, *Enterobacter* spp.  8.3%, *E. coli* | *bla*_KPC_  *bla*_VIM_  *bla*_KPC_ |
| TURIN  (no data at T0)  (no data at T1-T3) |  |  |  |  |
| BOLOGNA  (31 positives at T0)  (21 positives at T1-T3) | 87.1%, *K. pneumoniae*  3.2%, *K. pneumoniae*  3.2%, *K. pneumoniae*  3.2%, *E. coli*  3.2%, *Enterobacter* spp. | *bla*_KPC_  *bla*_NDM_  *bla*_KPC_*; bla*_NDM_  *bla*_NDM_  *bla*_VIM_ | 81.0%, *K. pneumoniae*  9.5%, *Enterobacter* spp.  4.8%, *K. pneumoniae*  4.8%, *K. pneumoniae* | *bla*_KPC_  *bla*_VIM_  *bla*_NDM_  *none* |
| FLORENCE  (18 positives at T0)  (2 positives at T1-T3) | 61.1%, *K. pneumoniae*  33.3%, *K. pneumoniae*  5.6%, *K. pneumoniae* | *bla*_KPC_  *bla*_VIM_  *bla*_KPC_*;bla*_VIM_ | 100%, *K. pneumoniae* | *bla*_KPC_ |
| CATANIA  (3 positives at T0)  (3 positives at T1-T3) | 100%, *K.pneumoniae* | *bla*_KPC_ | 100%, *K. pneumoniae* | *bla*_KPC_ |
| NAPLES  (10 positives at T0)  (3 positives at T1-T3) | 80.0%, *K. pneumoniae*  10.0%*,* *K. pneumoniae*  10.0%, *E. coli* | *bla*_KPC_  *none*  *bla*_KPC_ | 66.7%, *K. pneumoniae*  33.3%, *K. pneumoniae* | *bla*_KPC_  *none* |
| PALERMO  (12 positives at T0)  (16 positives at T1-T3) | 75.0%, *K. pneumoniae*  25.0%, *K. pneumoniae* | *bla*_KPC_  *bla*_KPC_*; bla*_OXA-48_-like | 75.0%, *K. pneumoniae*  25.0%, *Enterobacter* spp. | *bla*_KPC_  *bla*_VIM_ |

**Table 4.** Prevalence of carbapenem-resistant *Enterobacterales* (CRE) strains isolated in the various centres in wards different from Intensive Care Unit (non-ICU) in the observation period II at hospital admission (T0) and between week 1 and week 3 (T1-T3), Italy 2019-2020.

| **Period II** | **T0** | | **T1 – T3** | |
| --- | --- | --- | --- | --- |
| **no-ICU** | **Bacteria** | **Gene** | **Bacteria** | **Gene** |
| GENOA  (10 positives at T0)  (3 positives at T1-T3) | 80%, *K. pneumoniae*  10%, *E. coli*  10%, *Enterobacter* spp. | *bla*_KPC_  *bla*_KPC_  *none* | 66.7%, *K. pneumoniae*  33.3%, *K. pneumoniae* | *bla*_KPC_  *bla*_VIM_ |
| TURIN  (no data at T0)  (no data at T1-T3) |  |  |  |  |
| BOLOGNA  (11 positives at T0)  (15 positives at T1-T3) | 81.8%, *K. pneumoniae*  9.1%, *E. coli*  9.1%, *Enterobacter* spp. | *bla*_KPC_  *bla*_VIM_  *bla*_VIM_ | 80.0%, *K. pneumoniae*  6.7%, *K. pneumoniae*  6.7%, *E. coli*  6.7%, *Enterobacter* spp | *bla*_KPC_  *bla*_NDM_  *bla*_KPC_  *bla*_VIM_ |
| FLORENCE  (7 positives at T0)  (6 positives at T1-T3) | 85.7%, *K. pneumoniae*  14.3%, *Enterobacter* spp. | *bla*_KPC_  *bla*_VIM_ | 100 %, *K. pneumoniae* | *bla*_KPC_ |
| CATANIA  (70 positives at T0)  (8 positives at T1-T3) | 97.1%, *K. pneumoniae*  2.9%, *E. coli* | *bla*_KPC_  *bla*_NDM_ | 100%, *K. pneumoniae* | *bla*_KPC_ |
| NAPLES  (12 positives at T0)  (6 positives at T1-T3) | 58.3%, *K. pneumoniae*  16.7%*,* *K. pneumoniae*  8.3%, *E. coli*  8.3%, *E. coli*  8.3%, *Enterobacter* spp. | *bla*_KPC_  *bla*_OXA-48_-like  *bla*_VIM_  *none*  *bla*_NDM_ | 66.7%, *K. pneumoniae*  16.7%, *E. coli*  16.7%, *E. coli* | *bla*_KPC_  *bla*_KPC_  *None* |
| PALERMO  (85 positives at T0)  (48 positives at T1-T3) | 91.8%, *K. pneumoniae*  2.4%**,** *K. pneumoniae*  1.2%, *K. pneumoniae*  1.2%, *K. pneumoniae*  1.2%, *K. pneumoniae*  1.2%, *E. coli*  1.2%,*Enterobacter* spp.*+K. pneumoniae* | *bla*_KPC_  *bla*_KPC_*; bla*_NDM_  *bla*_OXA-48_-like  *bla*_VIM_  *bla*_KPC_*; bla*_OXA-48_-like  *bla*_KPC_  *bla*_KPC_*; bla*_VIM_ | 81.3%, *K. pneumoniae*  4.2%, *K. pneumoniae*  4.2%, *K. pneumoniae*  2.1%, *K. pneumoniae*  2.1%, *K. pneumoniae*  2.1%, *K. pneumoniae*  2.1%, *E. coli*  2.1%, *Citrobacter* spp. | *bla*_KPC_  *bla*_OXA-48_-like  *bla*_KPC_*; bla*_VIM_ *bla*_NDM_  *bla*_KPC_*; bla*_NDM_  *bla*_KPC_*; bla*_OXA-48_-like  *bla*_KPC_  *none* |

# Table 5. Statistical analysis of the proportion of carbapenem-resistant *Enterobacterales* (CRE) strains isolated at the various centres in Intensive Care Unit (ICU) and other wards (no-ICU) during the observation periods I (2018-2019) and II (2019-2020), at hospital admission (T0) and between week 1 and week 3 (T1-T3), Italy 2018-2020

| **Statistical analysis among Centres - Period I** | | |
| --- | --- | --- |
| **Wards** | **Centres with the highest proportion of CRE-positive patients**  **p-value (test type)** | **Centreswith the highest proportion of patients with *K. pneumoniae-****bla_KPC_*  **p-value (test type)** |
| ICU, T0 | <0.0001*(C)  Bologna(8%)**, p=0.0003(Z)  Palermo(10.5%)**, p<0.0001(Z) | 0.24(C) |
| ICU, T1-T3 | <0.0001*(C)  Turin(34.7%)**, p<0.0001(Z)  Palermo(27.3%)**, p<0.0001(Z) | 0.0003*(C)  Turin(100%)**, p=0.0088(Z) |
| no-ICU, T0 | 0.0013*(C)  Genoa(7.6%)**, p=0.0079(Z) | 0.36(C) |
| no-ICU, T1-T3 | <0.0001*(C)  Palermo(21.6%)**, p<0.0001(Z) | 0.37(C) |
| **Statistical analysis among Centres - Period II** | | |
| **Wards** | **p-value (test type)** | **p-value (test type)** |
| ICU, T0 | <0.0001*(C)  Catania(44.3%)**, p<0.0001(Z)  Palermo(23.1%)**, p<0.0001(Z) | 0.0008*(C)  Catania(100%)**, p=0.0213(Z) |
| ICU, T1-T3 | <0.0001*(C)  Palermo(54.9%)**, p<0.0001(Z) | 0.78(C) |
| no-ICU, T0 | <0.0001*(C)  Catania(43.2%)**, p<0.0001(Z) | 0.0012*(C)  Naples(58.3%)***, p<0.0001(Z) |
| no-ICU, T1-T3 | <0.0001*(C)  Palermo(20%)**, p<0.0001(Z) | 0.46(C) |
| **=significant statistical test; **=significant more frequent (p<0.05); ***=significant less frequent (p<0.05);C= chi-square test; Z= post hoc Z-test* | | |

This table shows the statistical results among the centres with the highest proportion of CRE-colonized patients (column 2) and of *K. pneumoniae* harbouring *bla_KPC_* (column 3) particularly.

Concerning period I, a significant colonization of CRE was observed in the ICU, in Bologna (8%, p=0.0003) and in Palermo (10.5%, p<0.0001) centres at the T0, as well as in Turin (34.7%, p<0.0001) and Palermo (27.3%, p<0.0001) centres during the hospitalization time (T1-T3).

A significant CRE colonization in Genoa (7.6%, p=0.0079) at T0, and in Palermo (21.6%, p<0.0001) was observed over the hospitalization time (T1-T3) in no-ICU.

During period II, at T0, the CRE colonization was significant in ICU for Catania (44.3%, p<0.0001) and Palermo (23.1%, p<0.0001) and only for Palermo (54.9%, p<0.0001) during the hospitalization time (T1-T3). The same two centres showed a significant CRE colonization presence in no-ICU, particularly in Catania (43.2%, p<0.0001) at T0, and during T1-T3 in Palermo (20%, p<0.0001).

When was evaluated the significant presence of *K. pneumoniae* harbouring *bla_KPC_,* it has been detected in Turin (100%, p=0.0088), as we can see in this table, considering the first period, in ICU during hospitalisation and in Catania (100%, p=0.0213), in the second period, at the admission; instead, significantly less frequent isolation of *K. pneumoniae* harbouring *bla_KPC_* in Naples (58.3%, p<0.0001) was observed among patients enrolled within no ICU at T0.

Supplementary figure

Fig.1

**Supplementary Figure 1.** Pergentages of the carbapenemase genes detected in North, Central, and South in Italy.

In supplementary figure 1 we reported the percentages considering three macro-area in Italy, i.e., North, Central, and South. Particularly, since the Central Italy included only one center, to reduce statistical bias for North and South area in which there are three centers, we performed the mean of the carbapenemase genes detected on three centers and subsequently we computed the percentage on all carbapenemase genes detected.

In addition in period I, we have observed for ICU centers one strain “gene no-detected” both at North and South area, in T1-T3 three strains gene no-detected” only in North area. In period II at T0 two strains “gene no-detected” were observed in South area.

For noICU centers, at T0 and T1-T3 were observed one strain gene no-detected both North and South area in period I, while in period II at T0 was observed one strain “gene no-detected” both North and South, while in T1-T3 two strains “gene no-detected” in South area only.
